# MADR-Net: multi-level attention dilated residual neural network for segmentation of medical images

**DOI:** 10.1038/s41598-024-63538-2

**Published:** 2024-06-03

**Authors:** Keerthiveena Balraj, Manojkumar Ramteke, Shachi Mittal, Rohit Bhargava, Anurag S. Rathore

**Affiliations:** 1 Yardi School of Artificial Intelligence, Indian Institute of Technology Delhi, Hauz Khas, New Delhi, 110016 India; 2 Department of Chemical Engineering, Indian Institute of Technology Delhi, Hauz Khas, New Delhi, India; 3 Department of Laboratory Medicine and Pathology, School of Medicine, University of Washington, Seattle, WA USA; 4 Departments of Bioengineering, Electrical and Computer Engineering, Mechanical Science and Engineering, Chemical and Biomolecular Engineering and Chemistry, Beckman Institute for Advanced Science and Technology, Cancer Center at Illinois, University of Illinois at Urbana-Champaign, Urbana, IL 61801 USA

**Keywords:** Deep learning, Semantic segmentation, Convolutional neural networks, Class-spatial attention module, Atrous convolution, Cardiology, Medical imaging

## Abstract

Medical image segmentation has made a significant contribution towards delivering affordable healthcare by facilitating the automatic identification of anatomical structures and other regions of interest. Although convolution neural networks have become prominent in the field of medical image segmentation, they suffer from certain limitations. In this study, we present a reliable framework for producing performant outcomes for the segmentation of pathological structures of 2D medical images. Our framework consists of a novel deep learning architecture, called deep multi-level attention dilated residual neural network (MADR-Net), designed to improve the performance of medical image segmentation. MADR-Net uses a U-Net encoder/decoder backbone in combination with multi-level residual blocks and atrous pyramid scene parsing pooling. To improve the segmentation results, channel-spatial attention blocks were added in the skip connection to capture both the global and local features and superseded the bottleneck layer with an ASPP block. Furthermore, we introduce a hybrid loss function that has an excellent convergence property and enhances the performance of the medical image segmentation task. We extensively validated the proposed MADR-Net on four typical yet challenging medical image segmentation tasks: (1) Left ventricle, left atrium, and myocardial wall segmentation from Echocardiogram images in the CAMUS dataset, (2) Skin cancer segmentation from dermoscopy images in ISIC 2017 dataset, (3) Electron microscopy in FIB-SEM dataset, and (4) Fluid attenuated inversion recovery abnormality from MR images in LGG segmentation dataset. The proposed algorithm yielded significant results when compared to state-of-the-art architectures such as U-Net, Residual U-Net, and Attention U-Net. The proposed MADR-Net consistently outperformed the classical U-Net by 5.43%, 3.43%, and 3.92% relative improvement in terms of dice coefficient, respectively, for electron microscopy, dermoscopy, and MRI. The experimental results demonstrate superior performance on single and multi-class datasets and that the proposed MADR-Net can be utilized as a baseline for the assessment of cross-dataset and segmentation tasks.

## Introduction

Biomedical imaging is an indispensable tool for applications such as localization of pathology, disease diagnosis, treatment planning, and disease management. The most commonly used non-interventional/invasive diagnostic mapping includes computed tomography, magnetic resonance imaging (MRI), digital mammography, fundus imaging, and other imaging modalities. These medical imaging tools offer the first line of choice due to their low cost, real-time functionality, and portability. Image formulation and reconstruction along with image processing and analysis are the two components of medical imaging^1^. Image formulation and reconstruction involve the process through which 2D and 3D images are typically formed from the projection data of an object. On the other hand, image processing involves enhancing the image properties to facilitate object identification and classification. Ease of image acquisition has paved the way for producing high-resolution images at extremely low cost^2^.

Computer-aided diagnosis for delineation of pathological structures is becoming an important tool for clinicians. Doctors use medical images to judge the condition of patients for clinical diagnosis. Medical image segmentation plays a crucial role in computer-aided diagnosis and intends to visualize the changes in the pathological or anatomical structure of the images. The segmentation techniques can be grouped into three different categories: (1) Manual segmentation, (2) Semi-automatic segmentation, and (3) Fully automatic segmentation. Manual segmentation is the initial step for determining the region of interest (ROI) and for precisely annotating the boundaries. Further, semi-automation techniques involve a user interface for initial ROI segmentation from the entire image. The fully automatic segmentation technique is based on supervised learning approaches, and they do not require any user interaction.

Currently, medical image segmentation is semi-automatic and suffers from various complications^3^: (1) The whole process needs to be carried out only by an experienced clinician, (2) The annotations will always include inter and intra-observer variability, and (3) This is a cumbersome and error-prone process that must be done for each patient individually. Consequently, automatic segmentation approaches have been developed to address these issues and facilitate higher patient throughput and lower inter-user discrepancy. However, automatic segmentation also suffers from a number of issues: (1) Low signal-to-noise ratio, (2) Poor contrast between the myocardium and blood pool, (3) Motion artifacts of the heart structures across patients and pathologies, (4) Brightness inhomogeneities, and (5) Low spatial and temporal resolutions^4^. Acknowledging the relevance and importance of segmentation tasks mentioned above, automation of this activity has been a major study topic of research in recent decades^5–14^.

Prior to the widespread application of deep learning, researchers utilized model-driven strategies (active contours, level sets, deformable models, and statistical shape models) for medical image segmentation, which typically require manual interventions. However, the emerging interest in computer-aided diagnosis (CAD) models aims to automate these processes for increased efficiency and accuracy. To achieve this objective, researchers proposed the encoder-decoder structure such as a fully convolutional network (FCN)^15^, U-Net^16^, and Deeplab^17^ which effectively automate image segmentation tasks. Badrinarayanan et al.^18^ proposed a deep convolutional encoder-decoder architecture that allows to perform pixel-level semantic segmentation. Ronneberger et al.^16^ extended Long et al.'s FCN^15^, by introducing the classical U-Net architecture, known for its end-to-end training ability and widespread application in the field of biomedical image segmentation. Although U-Net is effective, it has limitations such as loss of spatial information and difficulty in handling image variations.

Several variations of U-Net architectures, such as Attention U-Net^19^, Residual U-Net^20^, Multi Residual U-Net^21^, V-Net^22^, R2 U-Net^23^ and U2Net^24^, have been proposed in the literature. In this paper, while analyzing the strengths of U-Net architecture, we delicately examined the network architecture to identify a promising scope for future development. To extract features from different scales and sizes, we suggest replacing the convolution blocks of the classic U-Net with multi-dilated residual convolution blocks of rate d = 1, 3, 5, and 11 in this study. Embedding the ASPP module at the bottleneck position provides the network ability to efficiently capture multi-scale contextual information. Finally, the utilization of the channel-spatial attention mechanism played a pivotal role in extracting both channel-wise and spatial information, while suppressing noise and irrelevant features that are crucial for analysis.

In view of the above, we designed a novel deep learning architecture called deep multi-level attention dilated residual neural network (MADR-Net), that incorporates a class-spatial attention mechanism-driven decoder and inculcates the properties of dilated residual neural networks and atrous spatial pyramid pooling. Unlike the classic U-Net, MADR-NET extracts the features at different scales by integrating multiple dilated convolution modules. This process increases segmentation accuracy and captures multi-scale information.To extract the features at multiple scales and rich contextual details, atrous spatial pyramid pooling (ASPP) module with various dilation rates is used in the bottleneck layer of the encoder-decoder architecture. Further, channel-spatial attention modules are employed in skin pathways to suppress unnecessary areas in an input image while emphasizing important characteristics for a particular task. In this study, a hybrid loss function with a combination of cross-entropy, dice loss, and focal Tversky loss was introduced to enhance the models’ ability to quantify discrepancies between predicted and ground truth in medical image segmentation tasks. To validate the performance of the proposed MADR-Net architecture, four challenging clinical segmentation problems were addressed, namely echocardiogram, dermoscopy, electron microscopy, and MR images, demonstrating its efficacy across diverse medical imaging modalities. To validate the performance of the proposed architecture, we adopted three different architectures which include U-Net, Res Net, and attention U-Net. The main contributions of this proposed work are as follows:

- MADR-Net is based on the classical U-Net architecture of several biomedical imaging datasets. To overcome the loss of spatial information in U-NET, the convolutional block of U-Net is replaced by the multi-level dilated residual network.
- Channel-spatial attention modules are implemented to extract both shallow and deep feature maps, which increases the focus on the area of interest of target segmentation, resulting in the finest precision across all experiments.
- Based on our findings, hybrid loss function, speeds up the convergence and improves the performance of the segmentation task.
- MADR-Net has demonstrated its potential as a feasible and generalizable solution across various image modalities, including electron microscopy, dermoscopy, echocardiogram, and MRI, through its ability to segment medical tasks.
- The robustness of the proposed MADR-Net architecture is compared to recent state-of-the-art architectures such as U-Net, Attention U-Net, Multi residual U-Net, SU-Net, CNLU-Net and Res U-Net architectures.

In this paper, "Prior art" section discusses the related traditional and deep learning approaches used for the segmentation of various image modalities. "Network architecture" section highlights the network architectures, its components, and methodology. "Experimental setup" section presents the results and discussion in terms of ablation and comparison study. Finally, the conclusions of the study are presented in "Results and discussion" section.

## Prior art

### Traditional machine learning techniques

Traditional artificial intelligence approaches are based on computer-aided diagnosis systems that extract features from spatial, temporal, and morphological regions. However, the effectiveness of extracting these features is a real challenge in medical images due to their susceptibility to noise and motion artifacts. Medical image segmentation often depends on Level set^25^, multilevel thresholding^26^, Active shape models^27^, Active contour model^28^, Active appearance models^29^, Bottom-up method^30^, and Database-guided^31^. Bhandari et al.^32^ introduced different objective functions using cuckoo search to solve image segmentation through multilevel thresholding. Cheng and Wang^33^ presented homogram thresholding and region-based merging for color image segmentation. Ming-Ni Wu et al.^34^ proposed a color-based segmentation to track brain tumors using k-means clustering. Although several algorithms have been described and are successful in certain situations, segmentation of medical images remains one of the most difficult subjects in computer vision due to the complexity of feature representation.

### Deep learning techniques

Recently, deep learning approaches have demonstrated promising results in segmentation tasks due to their capacity to learn complicated characteristics from data^35^. Convolutional neural networks are commonly utilized for medical image processing applications such as localization, segmentation, and classification. Plain convolutional neural networks (CNN) have been extended to numerous networks, including Deep feedforward neural networks^36^, Deep long short-term memory (LSTM) architecture^37^, Inception CNN architecture^38^, Deep belief network (DBN)^39^, and Deep generative adversarial architecture (DGAN)^40^. Based on these, Fully convolutional network (FCN)^15^ and U-Net^16^ were proposed for semantic segmentation as they have exhibited remarkable performance. FCN architecture is trained end-to-end for pixel-wise prediction. U-Net architecture consists of two paths: encoder and decoder path. In the encoder or analysis part, deep features are learned and in the decoder part, segmentation is performed based on the learned features. These architectures have been applied to several 2D and 3D medical images for automated medical image assessment. Popular medical image segmentation tasks include brain tumor segmentation from magnetic resonance imaging, skin lesion segmentation from dermoscopy images, segmentation of the left ventricle from echocardiography images, and segmentation of the hippocampus region from electron microscopy images. Table 1 shows the visual comparison of the proposed MADR-Net with the baseline approaches with a detailed description of the encoder, decoder, bottleneck, and skip connection layers.

(i) *Segmentation of left ventricle, left atrium, and myocardial wall from Echocardiogram images:* Sarah Leclerc et al.^5^ introduced the largest publicly available dataset for 2D echocardiogram assessment. To get the best possible results on this dataset, a modified U-Net architecture was proposed, and the experts reached excellent agreement for the estimation of left ventricle end-diastolic (ED) and end-systolic (ES) with dice scores of 0.939 ± 0.043 and 0.916 ± 0.061, respectively. A lack of training data, low signal-to-noise ratio, and substantial variability among perspectives for collaborative learning are some of the limitations of echocardiogram data. To address these issues, Li et al.^41^ developed a multiview recurrent aggregation network (MV-RAN) for full cardiac cycle analysis. The MV-RAN architecture produced an average dice score of 0.92 ± 0.04 for the segmentation of the left ventricle. Liu et al.^42^ presented a deep learning model for the automatic segmentation of a 2D echocardiogram based on a deep pyramid local attention neural network. The correlation between the actual left ventricle ejection fraction and predicted left ventricle ejection fraction was 0.883 and 0.869, respectively for two different datasets, whereas the corresponding dice scores were 0.951 and 0.943 for ED and ES frames, respectively. Ali et al.^43^ proposed a fast and automatic deep learning framework by fusing Res Net and U-Net to enhance the segmentation results. Guo et al.^44^ presented a fusion of low-level and high-level features based on a spatial attention module along with a hybrid loss function.
(ii) *Segmentation of skin lesion from dermoscopy images*: The ISIC 2017 skin lesion analysis challenge was arranged by Bi et al.^45^, and the multiscale residual network took first place with an average Jaccard index of 79.40%. The segmentation of skin lesions remains a difficult process due to poor contrast, blurred boundaries, and varying sizes of cancer patches. To alleviate these drawbacks, Zhang et al.^46^ developed a deep supervised multi-scale network for the segmentation of skin lesions by designing a multi-scale connection block along with shallow and deep layers. The experiments were carried out on two different datasets and an average dice score of 87.5% and Jaccard index of 78.5% were reported. For the segmentation of skin lesions, Wei et al.^47^ suggested a novel attention-dense U-Net with adversarial training, which yielded a dice score of 0.8786 for the ISIC2017 dataset. Hasan et al.^48^ proposed a novel semantic segmentation network for robust skin lesion segmentation using depth-wise separable convolution instead of normal convolution to reduce the parameters of the network. To capture context characteristics and higher semantic feature information, Tang et al.^49^ presented a separable U-Net with stochastic weight averaging with an average dice coefficient of 86.93% and a Jaccard index of 79.26%.
(iii) *Brain tumor segmentation from magnetic resonance images*: Zhao et al.^50^ proposed a combination of a fully convolutional neural network along with conditional random fields (CRF) to segment the brain tumor. With 110 BRATS 2015 test cases, Kamnitsas et al.^51^ developed a dual pathway architecture built at multiple scales and obtained an average dice score of 0.847 for the automated segmentation of brain tumors. Havaei et al.^52^ explored different architectures based on CNN to segment glioblastomas i.e., low and high-grade MR images. To avoid overfitting in neural networks, Pereira et al.^53^ developed dropout, ReLu, and tiny convolution layers to discriminate between high and low-grade glioma images. In 2019, Wadhwa et al,^54^ observed that the combination of CRF with FCNN and CRF with DeepMedic is more successful in the segmentation of brain tumors.
(iv) *Segmentation of mitochondria from electron microscopy images*: Automatic segmentation of mitochondria and reconstruction in electron-microscopy images has proven to be a challenging task due to the variety of mitochondrial structures. Xiao et al.^55^ proposed a method for 3D mitochondria segmentation based on residual convolutional and highly supervised networks to overcome this problem. Manca et al.^56^ presented an automatic method for segmenting intracellular compartments such as mitochondria and endolysosomes. The authors compared the proposed algorithm with U-Net, V-Net, and DeepMedic algorithms and obtained dice scores of 0.855, 0.898, and 0.867, respectively. Oztel et al.^57^ reported encouraging findings for the autonomous segmentation of mitochondria in brain tissue using a deep convolutional neural network technique. Chacon et al.^19^ introduced a domain adaptation approach that relies on two coupled U-Net that share weights as well as a differentiable loss function that approximates the Jaccard index.

**Table 1:** Visual comparison of the proposed architecture with state-of-the-art techniques

Description of the Architecture								Encoder path		Bottleneck		Decoder path		Skip connection	
U-Net^15^: Encoder: A sequence of two convolution layers followed by max-pooling is used in the contracting path. 1 → 32 → 64 → 128 → 256 → 512 → 1024 Decoder: Up sampling was utilized in the expanded path, followed by convolution. 1024 → 512 → 256 → 128 → 64 → 32 → 1															
Attention U-Net^19^: Encoder: A sequence of two convolution layers followed by max-pooling is used in the contracting path. 1 → 32 → 64 → 128 → 256 → 512 → 1024 Decoder: Up sampling along with attention gates was utilized in the expanded path, followed by convolution. 1024 → 512 → 256 → 128 → 64 → 32 → 1															
Res U-Net^20^: Encoder: A sequence of two convolution layers with residual connection followed by max-pooling is used in the contracting path. 1 → 32 → 64 → 128 → 256 → 512 → 1024 Decoder: Up sampling was utilized in the expanded path, followed by convolution with the residual connection. 1024 → 512 → 256 → 128 → 64 → 32 → 1															
MADR-Net (Proposed): Encoder: A sequence of convolution layers with multi-scale residual connection followed by max-pooling is used in the contracting path. 1 → 32 → 64 → 128 → 256 → 512 → 1024 Decoder: The output of the encoder is fed into the PSP net and the output is fed into an expanded path. 1024 → 512 → 256 → 128 → 64 → 32 → 1															
	$3\times 3$ conv		Max-pooling		Addition		Residual block		Attention gates		Upsampling		Multi-scale residual block		Class-Spatial attention module

## Network architecture

**Figure 1:**
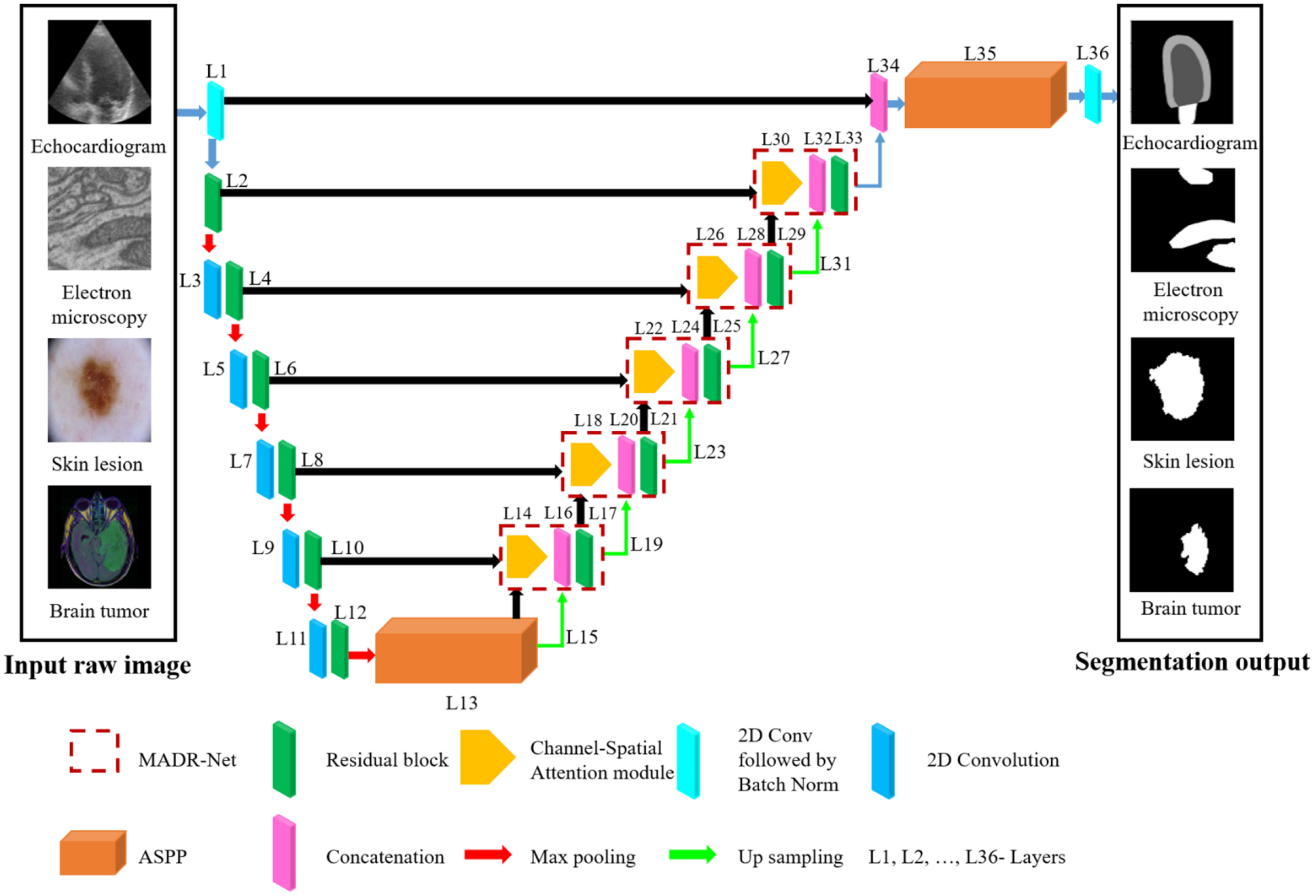
Schematic diagram of the proposed multi-level attention dilated residual neural network for the segmentation of medical image tasks.

In view of the shortcomings of the fundamental architecture, three important design enhancements were made. Fig. 1 depicts an overview of the proposed architecture. Inspired by U-Net^16^, Residual U-Net^58^, and ASPP^17^ architectures, a novel segmentation model named MADR-Net has been developed. The proposed MADR-Net architecture has 37 layers and takes advantage of the channel-spatial attention mechanism, ASPP, and multi-scale residual blocks. The multi-scale residual blocks help in tackling the vanishing gradient problem and build deeper neural networks to solve the degradation problem in each of the encoders.

Each encoder block consists of $3\times 3$ convolution blocks followed by multi-scale residual blocks and each multi-scale residual block has four parallel branches with a set of two stacked convolution layers such as batch normalization, ReLU, and 2D convolution layer. The outputs from the convolutional layers are added instead of concatenation to make the training earlier. An ASPP operator with four equal (1, 6, 12, 18) partitions has been used to bridge the encoder and decoder paths. In the decoder path, upsampling along with 2D convolution followed by batch normalization helps to improve the resolution of the convolutional feature^59^.

The output of the decoder block is sent through the ASPP block and depending on the segmentation task $1\times 1$ convolution with sigmoidal or softmax activation was applied. The different paths of the model notably the encoder, bottleneck, and decoder paths have been delineated as follows.

### Encoder path

Deep networks are naturally integrated with different levels of features (low/mid/high) and these features can be enriched by the depth or number of stacked layers. Driven by the significance of depth, the problem of vanishing grading could hamper the training process, and degradation problems may occur. To overcome these problems, He et al.^60^ introduced a deep residual learning framework that directly fits a desired underlying mapping instead of hoping for each stacked layer. Moreover, residual blocks are known to provide a deeper network and these connections facilitate model learning with reference to the input layer instead of learning from an unreferenced function.

**Figure 2:**
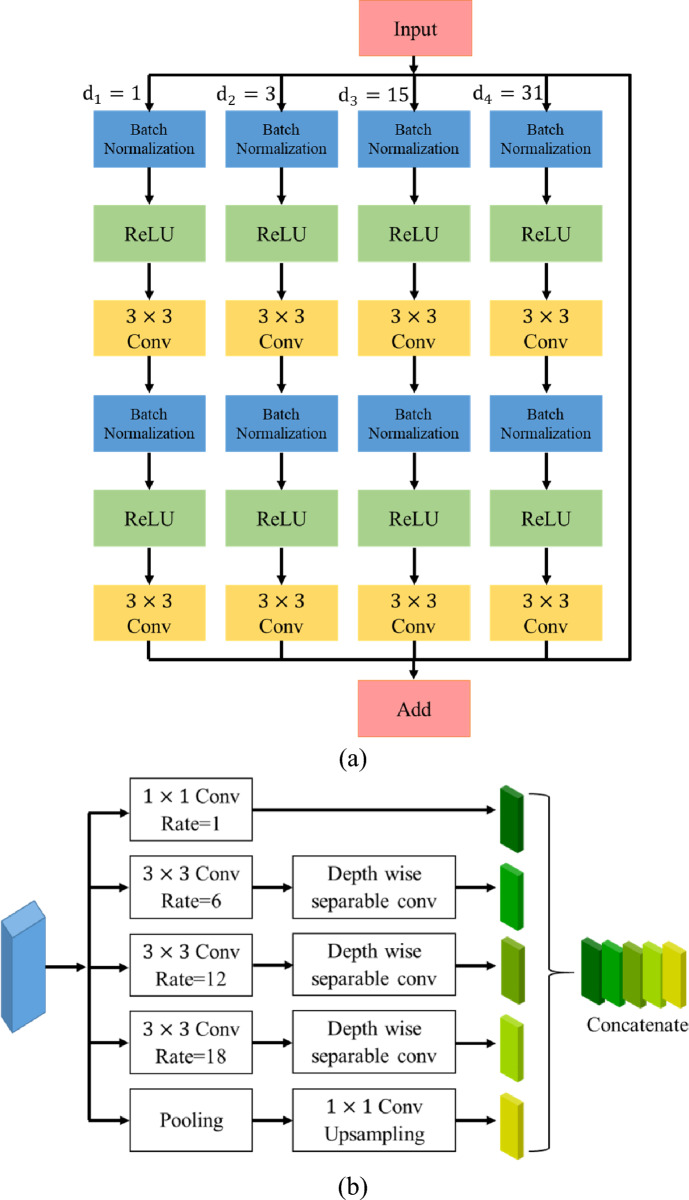
(**a**) Multi-level residual network with dilation rate = 1, 3, 15, and 31, and (**b**) Pyramid scene parsing pooling module.

The current U-Net architecture is a symmetric model and has only a few layers. We have replaced the basic U-Net architecture with the Multi-Dilated Residual blocks as shown in Fig. 2a. These residual blocks further contribute to feature propagation at both encoder and decoder paths. The general form of residual block can be expressed as

1 $$y_{N} = W_{s} \left( {x_{N} } \right) + {\mathcal{F}}\left( {x_{N} ,\left\{ {W_{N} } \right\}} \right)$$

2 $${x}_{N+1}=f({y}_{N})$$

Where ${x}_{N}$ and ${x}_{N+1}$ are the input and output with the Nth block respectively. ${\mathcal{F}}$ and f represent the residual mapping function and ReLU function. ${W}_{s}\left({x}_{N}\right)={x}_{N}$ which is an identity mapping. The performance of the residual block of Pre-ResNets is computed as follows

3 $$x_{N + 1} = W_{s} \left( {x_{N} } \right) + {\mathcal{F}}\left( {x_{N} ,\left\{ {W_{N} } \right\}} \right)$$

**Figure 3:**
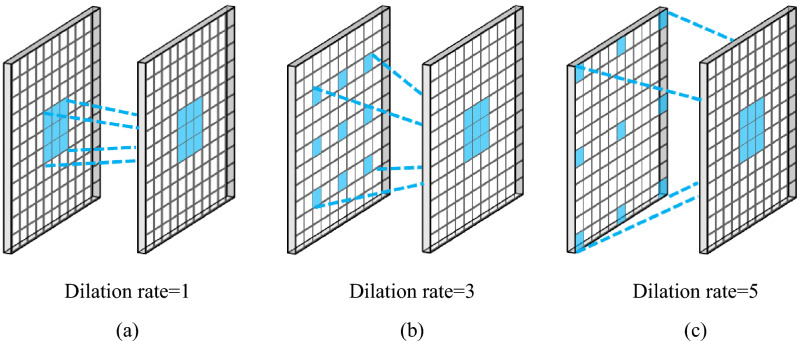
Convolution kernel of $3\times 3$ with different dilation rates.

In this study, the encoder path consists of 12 layers, which start with a 1 $\times 1$ convolution layer to avoid information loss, followed by a Multi-Dilated Residual block with an input size of $112\times 112\times 3$. Each multi-dilated residual block consists of $3\times 3$ parallel dilated convolutions at dilation rates of 1, 3, 5, and 11 along with the residual connection to extract features at different resolutions. Fig. 3 illustrates the 2D convolution with a spatial size of $3\times 3$ and different dilation rates (d = 1, 3, 5). In dilated convolution, the kernel with a $k\times k$ filter is amplified to $k+(k-1)(d-1)$. Normal convolution gets a $3\times 3$ receptive field, whereas dilated convolution with a dilation rate of 3 and 5 provides $5\times 5$ and $11\times 11$ receptive fields. Therefore, while normal convolution and dilated convolution have the same number of parameters, dilated convolution has a larger receptive field.

### Bottleneck

In the proposed approach, the atrous spatial pyramid pooling (ASPP)^17^ acts as the intermediary area that bridges the encoder and decoder paths. The ASPP module was developed to overcome the requirement of the constant size of the input image and resample features at multiple scales. The ASPP layer enlarges the field of view of the filters by capturing multiscale information precisely, as shown in Fig. 2b. In this study, multi-scale information can be captured through the ASPP module that consists of (1, 1) convolution followed by (3, 3) convolutions with different dilated rates (d = 6, 12, and 18) and a parallel max-pooling. The output of the bottleneck layers is sent through two separate paths: the convolutional transpose layer and the attention gate layer.

### Decoder path

The decoder path consists of 23 layers, each of which has a multi-dilated residual network, 2 $\times$ 2 convolutional transpose layer, skip connection, and Channel-spatial attention mechanism. Each decoder layer is coupled to the encoder layer by combining (L14, L10), (L18, L8), (L22, L6), (L26, L4), and (L30, L2) layers. Class-spatial attention module receives the output from the encoder layer and the preceding layer^9^. Initially, both average pooling (${F}_{avg}^{C}$) and max pooling (${F}_{max}^{C}$) operations are used to gather aggregate spatial information from the feature maps $({F}_{1}$). These spatial context descriptors are then forwarded to a shared network which consists of a multi-layer perceptron with one hidden layer to produce a channel attention map ${W}_{C}\in {\mathbb{R}}^{C\times 1\times 1}$. The channel attention is computed as:

4 $${W}_{C}(F)=\sigma \left({W}_{1}\left({W}_{0}\left({F}_{avg}^{C}\right)\right)+{W}_{1}\left({W}_{0}\left({F}_{max}^{C}\right)\right)\right)$$

Where ${W}_{0}\in {\mathbb{R}}^{C/r\times C}$ and ${W}_{C}\in {\mathbb{R}}^{C\times C/r}$ are the MLP weights that are shared for both inputs and *r* is the reduction ratio. In the spatial attention module (SAM), average pooled features (${F}_{avg}^{S}$) and max pooled features (${F}_{max}^{S}$) across the channel are concatenated and convolved by a standard convolution layer (${f}_{7\times 7}$) with the filter size of $7\times 7$ producing a spatial attention map ${W}_{S}\in {\mathbb{R}}^{1\times H\times W}$. The spatial attention is computed as:

5 $${W}_{S}(F)=\sigma \left({f}_{7\times 7}\left(\left[{F}_{avg}^{S};{F}_{max}^{S}\right]\right)\right)$$

6 $${F}_{1}={W}_{C}(F)\otimes F$$

7 $${F}_{2}={W}_{S}({F}_{1})\otimes {F}_{1}$$

**Figure 4:**
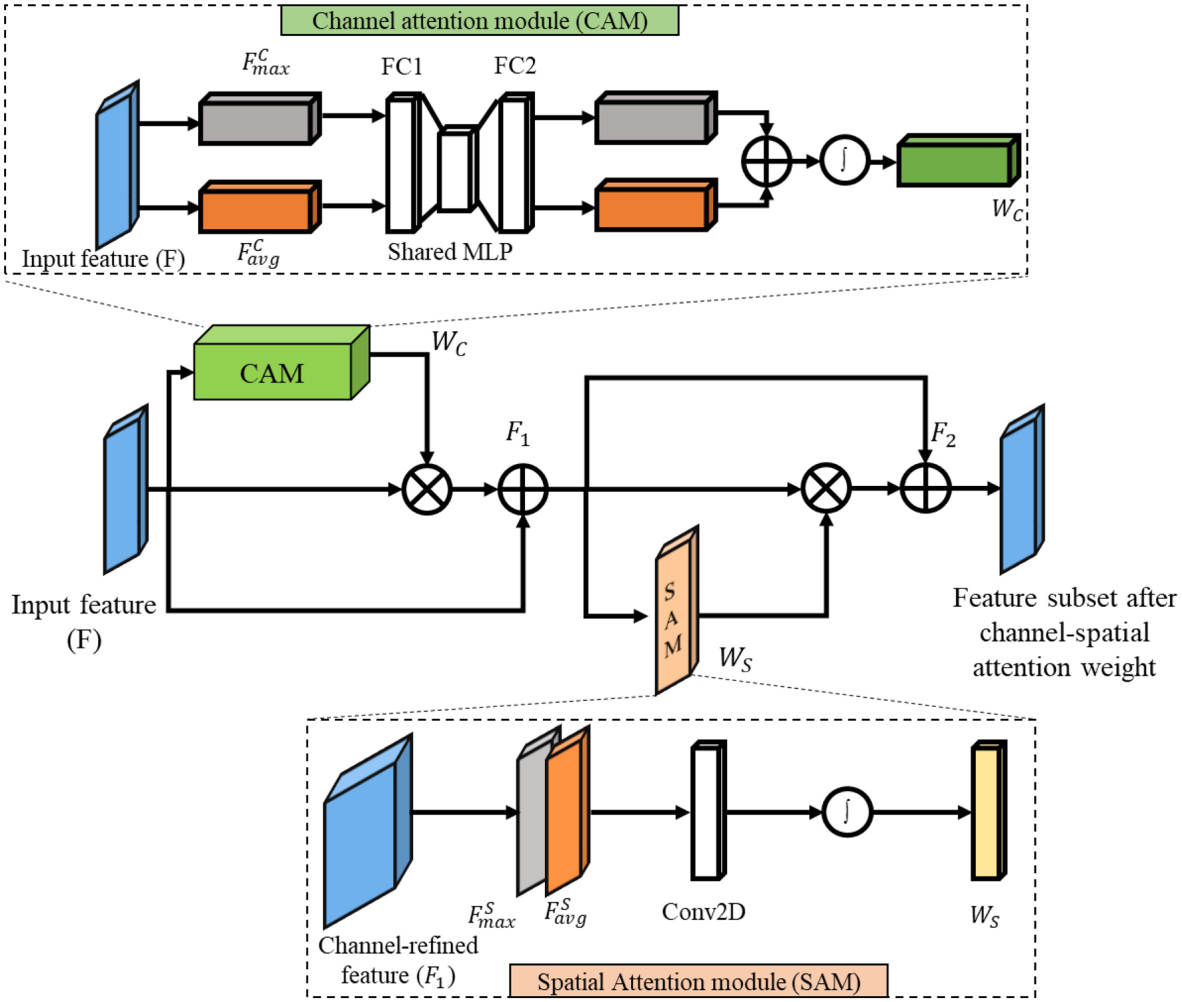
Architecture of channel-spatial attention mechanism.

Where $F\in {\mathbb{R}}^{C\times H\times W}$ is the intermediate feature and $\otimes$ represents element-wise multiplication. Finally, the attention block's output is concatenated with the preceding layer's upsampled output. Fig. 4, illustrates two sequential sub-modules: (1) channel attention module and (2) spatial attention module. At each and every convolutional block in deep networks, the intermediate feature map is adaptively improved by the channel-spatial attention module. The output is given to the residual block after concatenation, which is the same as the encoder path. Finally, the output of the multi-dilated residual block is combined with L1 and passed through ASPP architecture.

## Experimental setup

To evaluate the performance of the proposed deep multi-level attention dilated residual neural network (MADR-Net) architecture, we have tested it on four distinct medical image datasets. This section discusses the loss function used in the training process, dataset, and preprocessing steps involved in MADR-Net. Finally, the results of MADR-Net are compared with other state-of-the-art techniques on the different medical image datasets.

### Dataset

The effectiveness of the proposed MADR-Net architecture is evaluated with four publicly available datasets from different image modalities. Due to large variations in image sizes ranging from 256 $\times$ 256 to 1022 $\times$ 767 pixels, we rescalled all the data to 128 $\times$ 128 pixels. All datasets are normalized and to reduce the overfitting problem, different augmentation techniques were employed. Table 2 provides a full overview of the biomedical imaging dataset utilized in the proposed investigation.

- Cardiac Acquisitions for Multi-Structure Ultrasound Segmentation (CAMUS)^5^ consist of a two-dimensional apical two-chamber and four-chamber view sequence.
- Dermoscopy images were acquired from the Medical Image Computing and Computer-Aided Intervention (MICCAI)^61^ conference hosted by the International Skin Imaging Collaboration (ISIC) in 2017 for analysis of skin lesions.
- FIB-SEM^62^ dataset consists of serial section transmission electron microscopy (SSTEM) images acquired from the hippocampus region of the brain.
- Brain MR images with manual ground truth of fluid-attenuated inversion recovery (FLAIR) abnormalities were acquired from The Cancer Imaging Archive (TCIA)^63^.

**Table 2:** Biomedical imaging dataset used in this study

Dataset	Raw input image	Ground truth	Image modality	Segmentation task type	No. of Images	Resolution of the image
CAMUS (Echocardiogram)			Ultrasound	Multiclass	2000	549 $\times$ 778
ISIC-2017 (Skin Lesion)			Dermoscopy	Binary	2000	1022 $\times$ 767
FIB-SEM (Electron microscopy)			Electron microscopy	Binary	30 (1980 patches)	512 $\times$ 512
LGG (Brain tumor)			MRI	Binary	110	256 $\times$ 256

### Evaluation metrics

The performance of the segmentation model can be reliably evaluated in terms of dice similarity coefficient (DSC), Jaccard index or intersection over union (IoU), accuracy, sensitivity, and specificity. The disjoint sets are defined as follows: True positive (TP) set as $TP=GT\cap PR$, True negative (TN) set as $TN=\overline{GT }\cap \overline{PR }$, False positive (FP) set as $FP=\overline{GT }\cap PR$ and False negative (FN) set as $FN=GT\cap \overline{PR }$. In medical segmentation, the region of interest will be too small compared to the entire image, so TP will be low, and the background or non-infected region will be represented as TN^64^. This may lead to misleading performance, and so to overcome the class imbalance, it is necessary to focus on DSC and IoU metrics that robustly reflect the performance of the model.

8 $$DSC=\frac{2|GT\cap PR|}{\left|GT\right|+|PR|}$$

9 $$IoU=\frac{|GT\cap PR|}{|GT\cup PR|}$$

In the above equations, PR and GT are the predicted region and their corresponding ground truth. The measurements were graded from 0 (lowest) to 1 (highest). The performance metrics TN, TP, FN, and FP were used to evaluate accuracy, precision, and recall.

10 $$Accuracy=\frac{TP+TN}{(TP+TN+FP+FN)}$$

11 $$Precision=\frac{TP}{(TP+FP)}$$

12 $$Recall=\frac{TP}{(TP+FN)}$$

### Loss function

In our designed model, we used the combination of binary/categorical cross-entropy, dice loss, and focal Tversky loss to train the model. The binary cross-entropy (${{\ell}}_{BCE}$)^65^ loss function is computed as

13 $${{\ell}}_{BCE}=-\frac{1}{N}\sum_{i=1}^{N}{y}_{i}.log{\widehat{y}}_{i}+(1-{y}_{i})\text{log}(1-{\widehat{y}}_{i})$$

where ${\widehat{y}}_{i}$ is the *i*th scale value in the model output, ${y}_{i}$ is the corresponding target value and N is the output size. For the multi-class problem, the categorical cross-entropy loss function (${{\ell}}_{CCE}$) is computed as follows:

14 $${{\ell}}_{CCE}=-\frac{1}{N}\sum_{m=1}^{M}\sum_{i=1}^{N}{y}_{i}^{m}.log{\widehat{y}}_{i,m}$$

where ${\widehat{y}}_{i,m}$ is a matrix of predicted values for each class, where *i* and *m* iterate over all pixels and classes, respectively. Cross entropy loss is predicted by minimizing pixel-wise error, which results in poor quality segmentation of smaller ROI. Apart from the cross-entropy loss function, the Sorensen-Dice index^66^ was used for evaluating segmentation accuracy. The DSC can be defined in terms of the per-pixel classification of TP, FP, and FN which is given in Eq. (4). The dice loss (${{\ell}}_{DSC}$) can be defined as:

15 $${{\ell}}_{DSC}=1-DSC$$

On the other hand, the dice loss gradient is intrinsically unstable with unbalanced class data with a small denominator^67^. Focal Tversky (${{\ell}}_{FT}$)^68^ loss is defined as:

16 $${{\ell}}_{FT}=\sum_{m=1}^{M}{\left(1-\frac{\sum_{i=1}^{N}{p}_{0i}{q}_{0i}}{{\sum }_{i=1}^{N}{p}_{0i}{q}_{0i}+\alpha \sum_{i=1}^{N}{p}_{0i}{q}_{1i}+\beta \sum_{i=1}^{N}{p}_{1i}{q}_{0i}}\right)}^{\frac{1}{\gamma }}$$

where ${p}_{0i}$ and ${p}_{1i}$ are the probability of pixel *i* belonging to the foreground class and background class, respectively. ${q}_{0i}$ takes the value of 1 for the foreground and 0 for the background, whereas ${q}_{1i}$ is 1 for the background and 0 for the foreground. Here, we have chosen $\alpha =0.7 \text{and} \beta =0.3$ to improve recall in the case of large class imbalances. To overcome the segmentation of small ROI, the focal Tversky loss function with $\gamma =1.33$ was used to control the background and hard ROI. The hybrid loss adapts cross-entropy loss in combination with dice loss and focal Tversky losses to handle class imbalance.

### Run time

Experiments were performed in a server with an Intel(R) Silver(R) 4210 CPU on 2.19 GHz and 128GB RAM. The training time of the MADR-Net with the best performance was 14 hrs and 4 seconds per image for testing. The proposed architecture is optimized by Adam optimizer and started the training with a batch size of 16. The learning rate of the proposed algorithm is set to 1⨯10^−3^ which slowed down the convergence rate. The size of the images in each dataset is different, so the images are resized before feeding them to the model. We have utilized 80% data for training, 10% of the dataset for testing, and the remaining 10% for validation. All the models were trained for 500 epochs at a reduced learning rate to create a more generic model.

## Results and discussion

To validate the performance of the proposed MADR-Net architecture, we trained, validated, and tested the model on four different publicly available datasets (FIB-SEM, ISIC 2017, LGG, and CAMUS). In this section, the proposed architecture is compared with other baseline architectures such as U-Net^16^, Attention U-Net^20^, and Residual U-Net^58^ for the different segmentation tasks.

### Ablation study on the proposed MADR-Net on binary segmentation

The effectiveness of the proposed model is evaluated by binary segmentation of the infected part from the non-infected tissue. The influence of class distribution on the performance of semantic segmentation has been investigated. The segmentation performance of the proposed MADR-Net model is illustrated in Tables 3 and 4. Along with the performance measures, training times for all the architectures were noted for 500 epochs. As compared to other networks such as U-Net, Attention, and Residual U-Net, our proposed MADR-Net achieves an average improvement of 4.16%, 7.1%, and 1.75% (in terms of DSC), and 5.43%, 8.73% and 2.38% (on Jaccard) respectively for the FIB-SEM dataset. For ISIC 2017 dataset, the dice scores for U-Net, Attention U-Net, Residual U-Net, and MADR-Net are 87.08%, 86.64%, 88.14%, and 89.46% respectively. It can be seen that the dice score for MADR-Net outperforms the U-Net, attention U-Net, and residual U-Net by 2.74%, 3.43%, and 9.6%, respectively for the LGG dataset. Furthermore, the proposed algorithm offers comparable accuracy, precision, and recall on the test set of FIB-SEM, ISIC 2017, and LGG datasets for binary segmentation.

**Table 3:** Comparison of state-of-the-art architectures on binary segmentation.

	Architectures	Accuracy	Precision	Recall	Dice	Jaccard	Training time
FIB-SEM dataset	U-Net	97.31	94.10	91.58	89.85	84.60	5:02:17
	Attention U-Net	97.38	92.53	87.76	86.95	81.30	5:21:34
	Res U-Net	97.78	94.55	92.39	92.30	87.65	8:38:28
	Proposed algorithm	**98.30**	**95.43**	**94.02**	**94.05**	**90.03**	6:46:12
ISIC dataset	U-Net	95.99	92.34	85.02	87.08	78.01	4:34:04
	Attention U-Net	95.61	90.42	85.60	86.64	77.50	4:48:29
	Res U-Net	96.02	91.83	85.42	88.14	79.42	8:55:27
	Proposed Algorithm	**96.36**	**92.50**	**87.35**	**89.46**	**81.44**	7:37:30
LGG dataset	U-Net	93.64	86.67	82.93	83.75	73.79	10:32:00
	Attention U-Net	93.32	86.08	84.91	83.06	73.89	11:26:00
	Res U-Net	94.41	79.58	78.31	76.89	64.88	16:08:00
	Proposed	**94.75**	**87.62**	**85.62**	**86.49**	**77.71**	14:11:00

Significant values are in [bold].

**Table 4:** Comparison of state-of-the-art architectures on multi-class CAMUS dataset.

Architectures	Accuracy	Precision	Recall	Jaccard	Dice Epi	Dice endo	Dice LA	Training time
U-Net	95.24	95.25	95.22	91.23	94.96	92.42	**90.07**	7:36:26
Attention U-Net	95.35	95.35	95.36	91.61	95.16	92.27	89.31	8:04:28
Res U-Net	94.53	94.55	93.15	90.18	94.54	90.80	88.88	15:46:54
Proposed	**95.70**	**95.72**	**95.21**	**91.85**	**96.20**	**92.58**	89.90	13:01:55

Significant values are in [bold].

### Ablation study on the proposed MADR-Net on multi-class segmentation

Left ventricle endocardium (LV endo), left atrium (LA), and left ventricle epicardium (LV epi) gathered under one roof in the segmentation process and the proposed architecture was used to semantically segment all the three classes. Table 4 shows the comparison of the proposed MADR-Net with other state-of-the-art architectures on the CAMUS dataset for multi-class segmentation. The proposed algorithm yields a LV epi dice score of 96.20% and outperforms baseline architectures by 1.24%, 1.04%, and 1.66% for U-Net, Attention U-Net, and Residual U-Net, respectively. Similarly, the proposed architecture yields an average improvement of 0.16%, 0.31%, and 0.1785% (in terms of Endo DSC) for U-Net, Attention U-Net, and Residual U-Net. However, the maximum LA DSC is achieved by U-Net (90.07%) when compared to the proposed MADR-Net (89.90%). The maximum accuracy of 95.70% and minimum accuracy of 94.53% are obtained for the MADR-Net and Residual U-Net datasets, respectively. Precision and recall of 95.72% and 95.21% are obtained for the multiclass segmentation task.

### Performance and parameter comparison of different architectures

**Figure 5:**
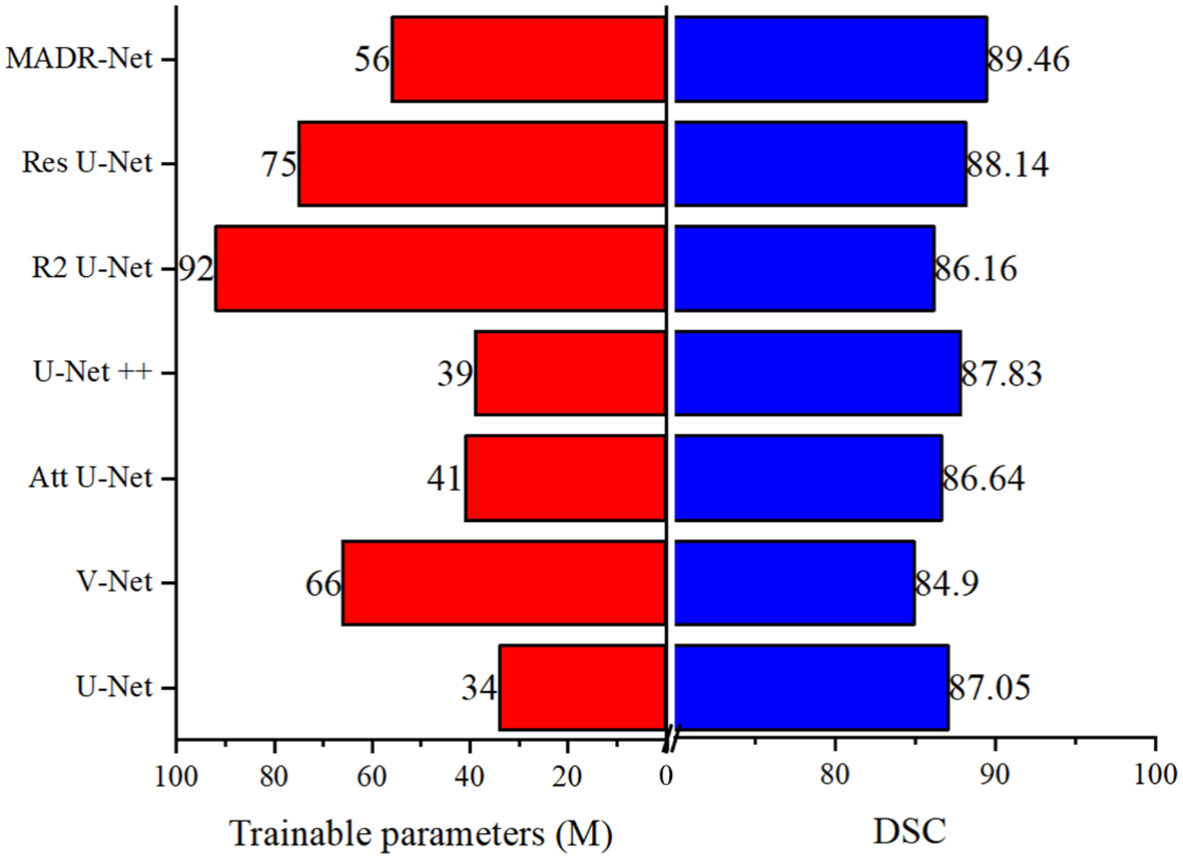
Relation between the performance and number of training parameters.

The size of the input image is 128 × 128 pixels and the state-of-the-art methods (U-Net, V-Net, Att U-Net, U-Net ++, R3 U-Net, and Res U-Net) are evaluated for different segmentation tasks with the following structure: 1 $\to$ 32 $\to$ 64 $\to$ 128 $\to$ 256 $\to$ 512 $\to$ 1024 $\to$ 512 $\to$ 256 $\to$ 128 $\to$ 64 $\to$ 32 $\to$ 1. Fig. 5 illustrates the number of training parameters and performance measures (DSC) for each segmentation technique on ISIC 2017 dataset. To provide a fair comparison, all the state-of-the-art techniques are replicated using the original code provided in their article, while maintaining the same preprocessing and training environment. Table 1S reports the parameter, inference time, and segmentation results obtained from ISIC 2017 dataset. To evaluate the effectiveness of the proposed MADR-Net method, we conducted a comparison with state-of-the-art techniques based on the number of parameters and DSC using 128 × 128 input image. However, the training time per each epoch is comparatively large depending upon the dataset. The training time for the proposed MADR-Net model took approximately 7 hours, while the testing time per image was 0.91 seconds. In addition, the proposed MADR-Net technique required a moderate number of trainable parameters which is 56M compared to 66M, 92M and 75M in the cases of V-Net, R2U-Net and Res U-Net architectures, respectively (see the supplementary Table 1S for further details). Overall, the proposed MADR-Net architecture has the potential to be viable in routine clinical settings due to its remarkable efficiency and superior segmentation performance.

### Visual inspection of feature maps

**Figure 6:**
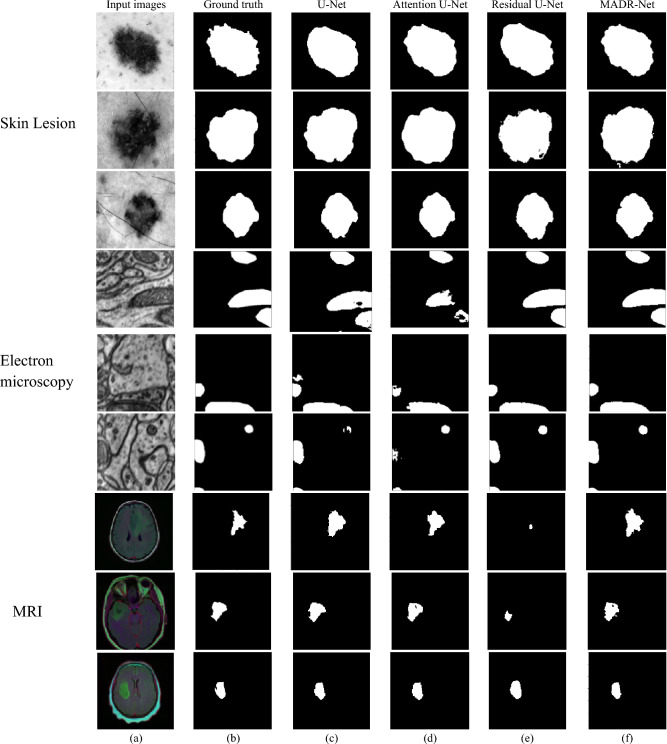
Results of single-class image segmentation: (**a**) input image, (**b**) Ground truth mask, (**c**) U-Net, (**d**) Attention U-Net, (**e**) Residual U-Net, and (**f**) MADR-Net.

**Figure 7:**
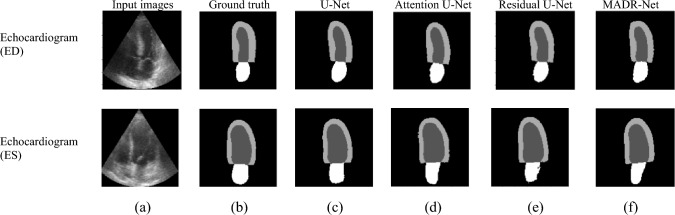
Results of multi-class image segmentation: (**a**) input image, (**b**) Ground truth mask, (**c**) U-Net, (**d**) Attention U-Net, (**e**) Residual U-Net, and (**f**) MADR-Net.

Using ablation experiments and quantitative evaluations to demonstrate performance differences may not be adequate to properly grasp the advantage and behavior of the proposed MADR-Net model. Tables 3 and 4 show the superior performance of the proposed architecture. Fig. 6 depicts the segmentation results of skin lesion, electron microscopy, and MRI with different baseline architectures. The study also focused on the comparison of different architectures in terms of convergence. Figs. 6 and 7 visualize the segmentation output of single-class and multi-class segmentation with different baseline architectures. Fig. 7a and b illustrate the raw input images and their corresponding ground-truth masks of the echocardiogram image with ED and ES frames. Fig. 7c–f represents the predicted segmentation masks of U-Net, Attention U-Net, and Residual U-Net.

**Figure 8:**
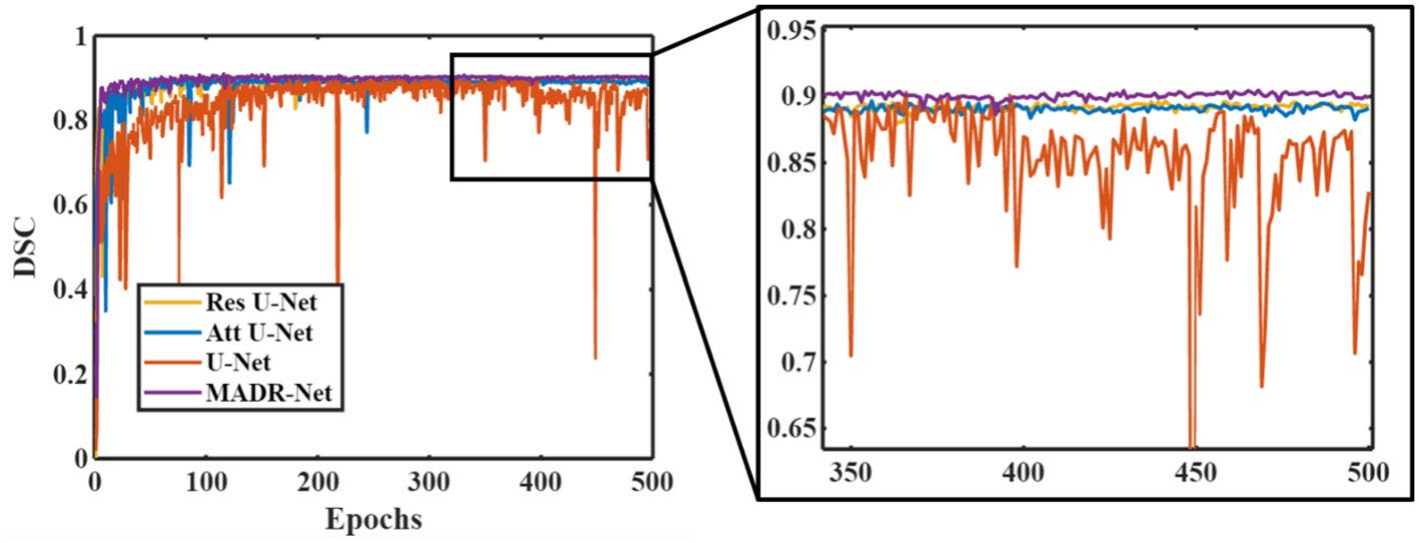
Progression of the validation dice score with respect to the number of epochs for Skin cancer dataset.

Fig. 8 depicts the mean DSC value for each of the networks. It can be observed that, while the majority of the baseline architecture generates results that are similar, the convergence of the proposed MADR-Net is quicker than other architectures for the segmentation of the various datasets. This is due to the interaction of multi-scale residual blocks and ASPP networks, which allows the MADR-Net to achieve pixel-perfect segmentation (see the supplementary Figs. 1S and 2S for further details).

**Figure 9:**
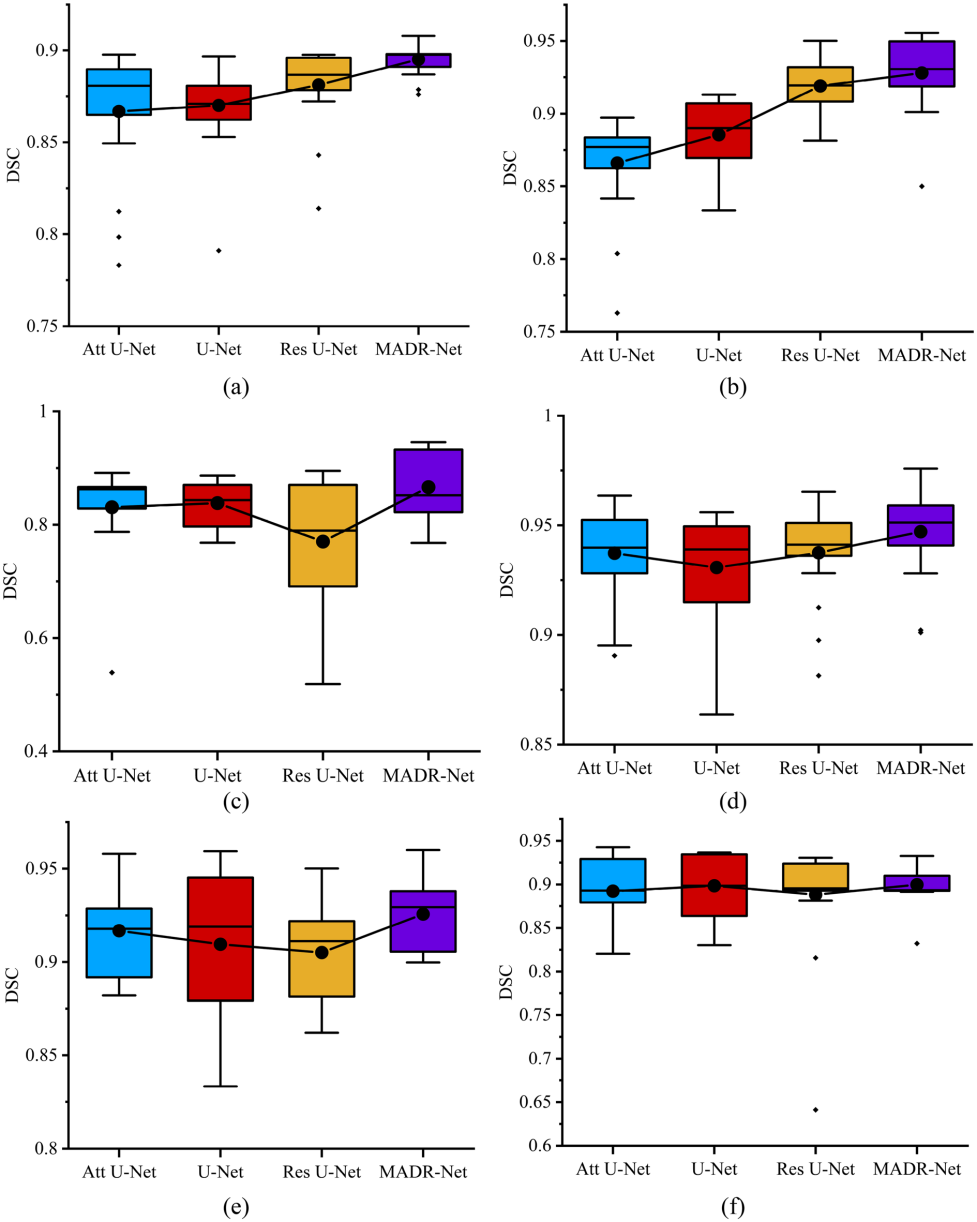
Box plot of test dataset with respect to DSC: (**a**) Test dice score for skin cancer dataset, (**b**) Test dice score for Electron Microscopy dataset, (**c**) Test dice score for MRI dataset, and (**d**, **e**) Test dice score for Echocardiography dataset with respect to LV, Myo, and RV.

Test results for four different architectures such as U-Net, Attention U-Net, Residual U-Net, and proposed MADR-Net are summarized in Fig. 9. The proposed algorithm outperforms the state-of-the-art techniques with an average dice score of 89.50%, which is 3% and 2% greater than Attention U-Net and U-Net, respectively, for skin lesion segmentation as shown in Fig. 9a. From Fig. 9b, it is observed that an average dice score of 97.31%, 97.83%, 97.78%, and 98.30% are obtained for Attention U-Net, U-Net, Res U-Net and MADR-Net architecture for the Electron microscopy dataset. Figure 9c depicts the performance of the proposed MADR-Net architecture with an increase of 4%, 4%, and 14% dice score with respect to Attention U-Net, U-Net, and Res U-Net. Dice scores are observed to be significantly higher for the CAMUS dataset containing LV, Myo, and RV in Fig.9d–f. As a result, we have concluded that MADR-Net is superior to U-Net in terms of both its capacity for learning and its potential for generalization.

## Discussion

As discussed above, to validate the performance of the proposed MADR-Net architecture, experiments were conducted for several classes of medical images which have their own set of challenges. It must be emphasized that in all circumstances, the proposed architecture achieves convergence substantially faster. However, empirical observation revealed that after introducing the ASPP and class-spatial attention module along with the multiscale residual block, the model was more effective in precise distinguishing of edges and texture of images, particularly in dermoscopy and electron microscopy images. In the electron microscopy dataset, the region of interest being segmented spans the majority of the image, and thus there is a tendency to over-segment the images. In U-Net architecture, the contextual information is passed through the skip connection which acts as a bridge between the encoder and decoder path. Further, to enhance the learning capability, non-linear multi-scale residual blocks were used to jump over multiple layers and to restore the information loss while concatenating features from contraction and expansion paths.

**Figure 10:**
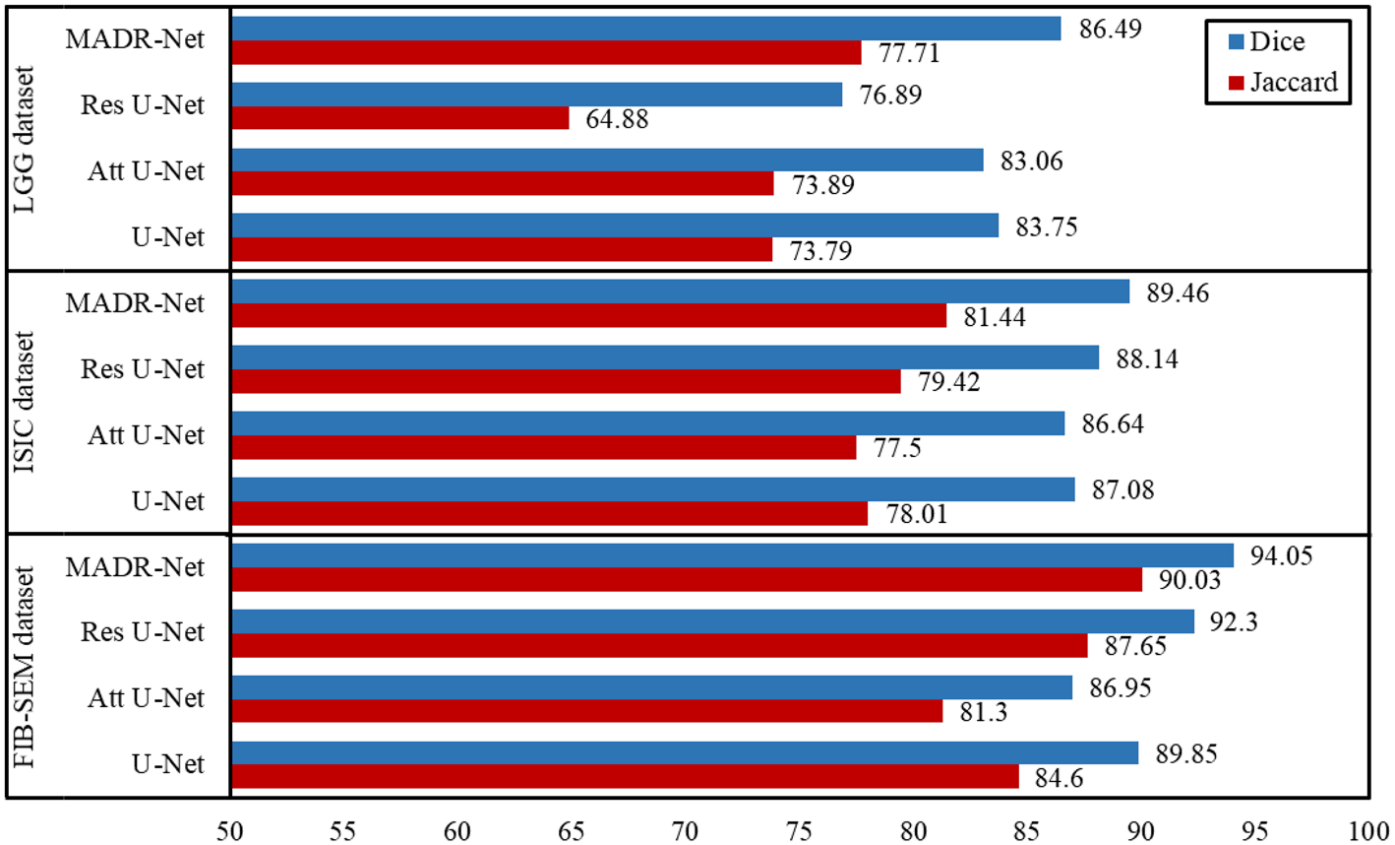
Comparison of DSC and Jaccard based on different methods on three different datasets.

To improve the segmentation accuracy over U-Net, the multi-level dilated residual block was added to the U-Net architecture to create a multi-level dilated residual U-Net. In addition, class-spatial attention modules were also introduced to the multi-level dilated residual U-Net in order to improve the segmentation quality in terms of DSC and Jaccard index. Fig. 10 depicts the performance of the proposed architecture with three state-of-the-art techniques such as U-Net, Res U-Net, and Att U-Net on three different datasets. More specifically, MADR-Net has a relative improvement of 5.43%, 8.73%, and 2.38% for U-Net, Attention U-Net, and Residual U-Net in terms of the Jaccard index by using the FIB-SEM dataset. MADR-Net has a relative improvement of 3.43%, 3.92%, and 2.02% for U-Net, Attention U-Net, and Residual U-Net respectively in terms of the Jaccard index for the ISIC-2017 dataset. Similarly, for the LGG MRI dataset, MADR-Net offered an improvement of 3.92%, 3.82%, and 12.83% Jaccard index for U-Net, Attention U-Net, and Residual U-Net, respectively. The experimental results demonstrate that the proposed MADR-Net has significant potential to offer robust performance in evaluating single-class segmentation. For multi-class segmentation, the CAMUS dataset was used and an improvement of 1.24%, 1.04%, and 1.66% for Epi, 0.16%, 0.32%, and 1.78% for Endo has been obtained by using U-Net, attention U-Net, and residual U-Net, respectively, in terms of dice score.

**Figure 11:**
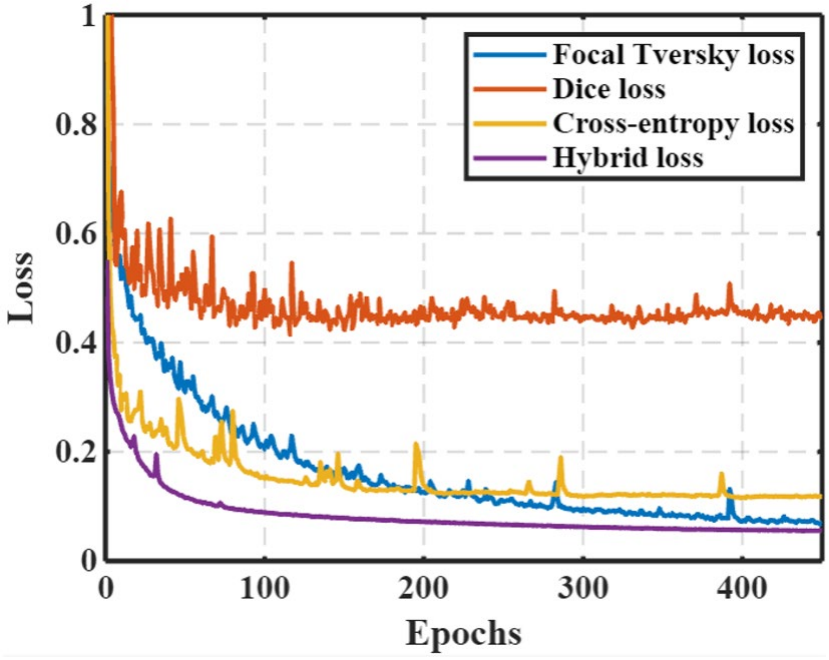
Loss function plot trained on the ISIC-2017 dataset for the MADR-Net architecture for the selected loss functions, i.e. Dice loss, Focal Tversky loss, cross-entropy loss and hybrid loss.

The model was trained on different loss functions, for example, dice loss, cross-entropy loss, focal Tversky loss, and a combination of these losses. From Fig. 11, we have observed that the model achieves higher DSC for all loss functions except for the dice coefficient loss function. Furthermore, exploratory research was conducted on how the number of filters, batch size, optimizers, and loss function impact the outcome. A remarkable improvement in segmentation was observed in both single and multi-class segmentation approaches. Results suggest the presence of an attention mechanism, multi-scale residual block, and ASPP network improves the retention of spatial and contextual information, which is often lost during the concatenation of features. When compared to U-Net, the proposed encoder-decoder architecture enhanced the segmentation of images.

In addition to examining the best performance model for each run, we also analyzed the model performance over each epoch. The performance analysis of the validation data on each epoch was reported in Fig. 8. Furthermore, for the four different datasets, a relative improvement of 4.2%, 3.43%, and 3.92% was observed by using MADR-Net over U-Net. Another striking observation is that except for some minor fluctuations in the validation process, the standard deviation of the proposed MADR-Net is substantially lower and thus demonstrates the dependability and robustness of the model. Table 5 offers a comparison of literature findings in terms of DSC and Jaccard. It is evident that the proposed algorithm outperforms all other state-of-the-art-architectures for both single and multi-class segmentation.

**Table 5:** Experimental results of proposed approaches for different datasets and comparison against other state-of-the-art approaches

Dataset	State-of-the-art architectures		Performance metrics	
	References	Proposed model	DSC	Jaccard
FIB-SEM	Ibtehaz et al.^21^	Multi residual U-Net	–	87.94
	Xiao et al.^69^	Deep contextual residual network	94.7	90.00
	Mekuc et al.^12^	Deep neural network (HighRes3DZMNet) and active contours	92.60	–
	Meyer et al.^13^	U-Net	93.70	–
	Shuvo et al.^14^	Classifier and Localizer model (CNL U-Net)	92.82	86.68
	**Proposed model**	**MADR-Net**	**94.05**	**90.03**
ISIC-2017	Zhang et al.^46^	Deep-supervised multiscale network	87.50	78.50
	Goyal et al.^70^	Ensemble deep learning	87.10	79.30
	Bi et al.^45^	Deep residual network	–	79.40
	Hasan et al.^48^	Depth-wise separable convolution	–	77.5
	Farahani et al.^10^	Small and Reduced U-Net (SU-Net)	84.44	–
	Lama et al.^11^	U-Net with squeeze-and-excitation residual	88.00	80.70
	Su et al.^71^	MSU-Net	86.50	77.1
	Shuvo et al.^14^	Classifier and Localizer model (CNL U-Net)	84.21	73.59
	**Proposed model**	**MADR-Net**	**89.46**	**81.44**
LGG	Ibtehaz et al.^21^	Multi residual U-Net	–	78.19
	Zhao et al.^50^	Fusion of FCNN and Conditional random field	84.00	–
	Naser et al.^72^	U-Net	84.00	–
	Shuvo et al.^14^	Classifier and Localizer model (CNL U-Net)	86.76	77.29
	**Proposed model**	**MADR-Net**	**86.49**	**77.71**
CAMUS	Chao et al.^73^	Segment Anything Model	86.00	75.60
	Leclerc et al.^74^	Refining U-Net	92.10 (Endo) 94.80 (Epi)	–
	Upadhyay et al.^75^	VGG16 U-Net	94.12 (Endo) 87.86 (Epi) 90.20 (LA)	–
	Sfakianakis et al.^76^	Geometrically constrained Ultrasound data augmentation in U-Net	94.60 (Endo) 96.0 (Epi) 89.40 (LA)	–
	**Proposed model**	**MADR-Net**	**92.58 (Endo)** **96.20 (Epi)** **89.90 (LA)**	**91.85**

Significant values are in [bold].

## Conclusions

In this study, we have proposed an extended version of U-Net named multi-level attention dilated residual neural network (MADR-Net) for the segmentation of medical images. By replacing the U-Net architecture, high-level features were extracted from multiple receptive fields and these connections improve the generalizing capability of the residual learning. The contextual features were effectively used for the segmentation of the tumor region by leveraging the inherent property of multi-scale residual blocks and the ASPP network. On the other hand, the channel spatial attention mechanism is of significance as it enables the retention of intricate features throughout the training process by capturing both local and global features. We have evaluated the effectiveness of the proposed architecture on four different datasets, which further highlights the superiority of our network in comparison to baseline models.

Among the handful of publicly accessible datasets, we specifically selected those that exhibit the most unique challenges. ISIC 2017 dataset focused on skin lesion analysis which encounters challenges due to the presence of hair strands, which makes the model difficult to delineate skin lesions and the background. The electron microscopy (FIB-SEM) dataset presents challenges for segmentation due to the existence of artifacts and intricate biological features. There is a limited number of samples for the CAMUS dataset, which consists of three classes with 2-chamber and 4-chamber projections of the heart. The MRI dataset consists of T1 pre-contrast, FLAIR, and T1 post-contrast MRI sequences and tumor segmentation is challenging due to the high variability of tumor structure and location. U-Net architecture has set a benchmark for above mentioned medical image segmentation tasks. However, for complex images that are affected by artifacts and lack clear boundaries, the MADR-Net architecture dramatically enhances performance. More specifically, from Tables 3 and 4, it is observed that the performance of MADR-Net has improved by 6%, 3%, 4%, and 2% in comparison with U-Net architecture.

Furthermore, the proposed algorithm consistently outperforms baseline architectures such as attention U-Net, and Residual U-Net for MRI, ISIC 2017, FIB-SEM, and CAMUS datasets in terms of accuracy, precision, recall, Jaccard, and dice score. The proposed MADR-Net segmentation approach not only received a higher score in the assessment metric but was also visually more comparable to the ground truth masks. We investigated the performance of several flavors of the loss function and introduced a novel loss function that combines cross-entropy, dice loss, and focal Tversky loss. By using this loss function, training convergence is quick and well-behaved under the presence of an imbalanced dataset. In the future, we anticipate integrating the proposed MADR-Net model with domain-specific experts by coupling it with the post-processing stage to design better segmentation techniques over a wide range of applications.

### Supplementary Information

Supplementary Information.

## Data Availability

This study has been conducted using publicly available data and it can be accessed using the provided URLs: 1. CAMUS dataset: https://www.creatis.insa-lyon.fr/Challenge/camus/, 2. ISIC dataset: https://challenge.isic-archive.com/data/, 3. Electron Microscopy dataset: https://www.epfl.ch/labs/cvlab/data/data-em/, 4. LGG dataset: http://cancergenome.nih.gov/abouttcga/policies/informedconsent. We preprocessed the data.

## Code availability

Code of this work is available on GitHub through this link: https://github.com/COECBT/MADR-Net.
