# Supplementary material for: MADR-Net: multi-level attention dilated residual neural network for segmentation of medical images
Source: Sci Rep. 2024 Jun 3;14:12699. doi: 10.1038/s41598-024-63538-2 (PMC11148105; doi:10.1038/s41598-024-63538-2)
Supplement: Supplementary file 1 — Supplementary Information. [file 41598_2024_63538_MOESM1_ESM.docx]

**MADR-Net: Multi-level Attention Dilated Residual Neural Network for Segmentation of Medical Images**

Keerthiveena B^1^, Manojkumar Ramteke^1,2^, Shachi Mittal^3^, Rohit Bhargava^4^, and Anurag S. Rathore^1,2,^*

^1^Yardi School of Artificial Intelligence, Indian Institute of Technology Delhi, India.

^2^Department of Chemical Engineering, Indian Institute of Technology Delhi, India.

^3^Department of Laboratory Medicine and Pathology, School of Medicine, University of Washington, Seattle, WA, USA

^4^Departments of Bioengineering, Electrical & Computer Engineering, Mechanical Science & Engineering, Chemical and Biomolecular Engineering and Chemistry, Beckman Institute for Advanced Science and Technology, Cancer Center at Illinois, University of Illinois at Urbana-Champaign, Urbana, IL 61801 USA

Corresponding to:

Anurag Singh Rathore, Professor, Department of Chemical Engineering, Joint Faculty, Yardi School of Artificial Intelligence, Indian Institute of Technology Delhi, Hauz Khas, New Delhi, 110016, India, Phone +91-9650770650, Email: [asrathore@biotechcmz.com](mailto:asrathore@biotechcmz.com)

| 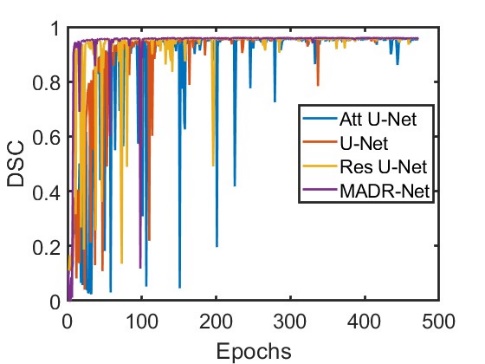 |
| --- |
| (a) |
| 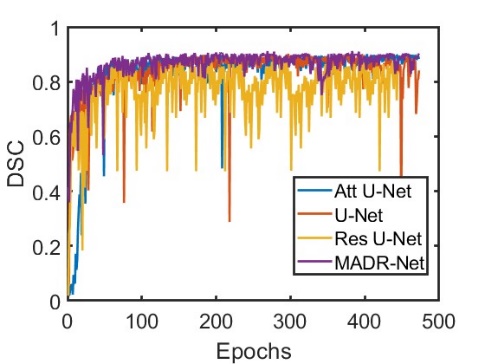 |
| (b) |

**Fig. 1S.** Progression of the validation dice score with respect to the number of epochs. (a) Validation dice score for Electron Microscopy dataset, (b) Validation dice score for MRI dataset

| 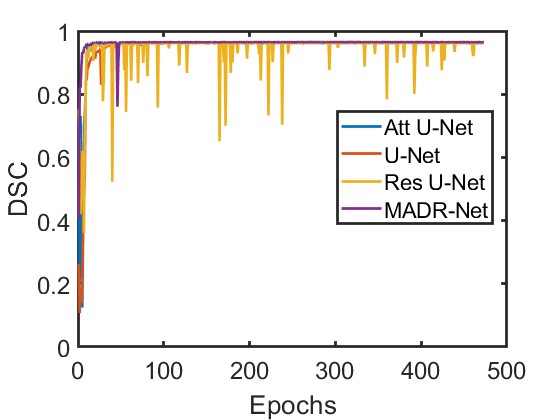 |
| --- |
| (a) |
| 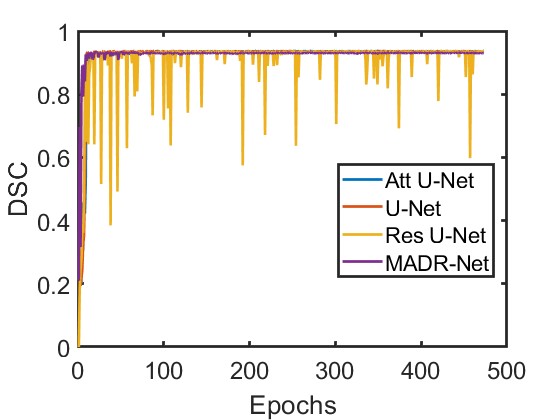 |
| (b) |
| 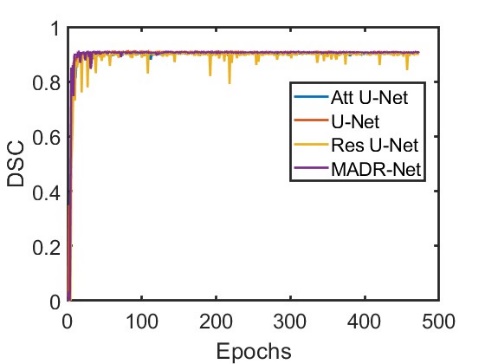 |
| (c) |

**Fig. 2S.** Progression of the validation dice score with respect to the number of epochs. (a)-(c) Validation dice score for Echocardiography dataset with respect to LV, Myo, and RV.

**Table 1S.** Architecture and number of network parameters

| Architecture | Number of parameters | Training Time (minutes) | Testing Time/Image (sec) | DSC |
| --- | --- | --- | --- | --- |
| U-Net | 34,607,394 | 274 | 0.54 | 87.05 |
| V-Net | 66,945,922 | 566 | 1.36 | 84.9 |
| Att U-Net | 41,072,759 | 288 | 0.57 | 86.64 |
| U-Net ++ | 39,751,980 | 282 | 0.65 | 87.83 |
| R2 U-Net | 92,671,554 | 646 | 1.79 | 86.16 |
| Res U-Net | 75,516,129 | 535 | 1.07 | 88.14 |
| MADR-Net | 56,733,598 | 457 | 0.91 | 89.46 |
